# Cervical cancer screening uptake and associated factors among Women Living with *Human Immunodeficiency Virus* in public hospitals, eastern Ethiopia

**DOI:** 10.3389/fonc.2023.1249151

**Published:** 2023-10-27

**Authors:** Dagnachew Tesfaye, Fitsum Weldegebreal, Firayad Ayele, Merga Dheresa

**Affiliations:** ^1^ Student Clinic, Haramaya University, Haramaya, Oromia, Ethiopia; ^2^ School of Medical Laboratory Sciences, College of Health and Medical Sciences, Haramaya University, Harar, Ethiopia; ^3^ School of Public Health, College of Health and Medical Sciences, Haramaya University, Harar, Ethiopia

**Keywords:** cervical cancer, screening, HIV, adult women, Ethiopia, WLHIV

## Abstract

**Background:**

Cervical cancer, the second leading cancer in Ethiopia women, is six times higher among women infected with the Human Immune Virus 1-infected women. Its screening provides protective advantages, and is linked to a decrease in the incidence of invasive cervical cancer and mortality. Although cancer screening has great advantages for early treatment and prevention of further complications, cervical cancer screening uptake is low among women in developing countries. Cervical cancer screening uptake among Women Living with Human Immunodeficiency Virus (WLHIV) is not well known in Eastern Ethiopia. Thus, we aimed to assess cervical cancer screening uptake and its associated factors among WLHIV in public hospitals in Harar, eastern Ethiopia.

**Methods:**

An institution-based cross-sectional study was carried out on 412 randomly selected HIV-positive women from March 20 to April 20, 2022. The results of the study were presented descriptively in percentages and analytically in odds ratio. Bivariate and multivariable logistic regression analyses were used to determine the presence and degree of association between dependent and independent variables. In the multivariable logistic analysis, a p-value of 0.05, and an adjusted odds ratio with a 95% confidence interval were considered to determine independent predictors for the uptake of cervical cancer screening.

**Results:**

Cervical cancer screening uptake among WLHIV was 57.5% (95% CI: 52.5, 62.9%). The uptake of cervical cancer screening was significantly associated with age between 20-29 years (AOR = 7.33; 95% CI: 1.98, 27.1), 40-49 years (AOR = 4.37; 95% CI: 1.48, 12.89), tertiary level of education (AOR = 0.197; 95% CI: 0.041, 0.946), good knowledge (AOR = 3.591; 95% CI: 2.123, 6.073), and monthly income of 2501(45.52 $) and above Ethiopian Birr (AOR = 0.389; 95% CI: 0.158, 0.959).

**Conclusions:**

More than half of the participants had undergone cervical cancer screening. Age, marital status, educational status, monthly income, and awareness of cancer screening uptake were all factors related to cervical cancer screening. To maximize uptake, it is necessary to create specific counseling and education programs that target HIV-positive women.

## Introduction

Cervical cancer is the fourth most frequent illness among women in the globe, with 604,000 new cases and 342,000 deaths expected in 2020 (1). It is a worldwide public health concern, with the highest prevalence ranging from 43.3 to 69.8 per 100,000 women (2) with over 90% of these occur in developing countries (3). Therefore, Sub-Saharan Africa had the greatest prevalence of cervical cancer among the top 20 burden countries in the world in 2018 (4). In Ethiopia, cervical cancer is the second most significant cause of sickness and death (5). Cervical cancer kills 5338 of the 7445 women diagnosed with it (6).

A combined burden of communicable and non-communicable diseases (7) as well as difficulties in providing preventative health services contribute to the high rate of cervical cancer in Africa (8–10). Lack of healthcare providers, barriers to treatment, and low public and professional awareness of cervical cancer attributed to low uptake of cervical cancer screening (11, 12).

HIV infection increases the risk of cervical cancer by six times among HIV-positive women than among their counterparts. In addition, human immune viruses are associated with several enabling factors for cervical cancer, including multiple sexual partners, early sexual debut, economic status, and smoking (1, 10, 11). Because of their susceptibility to cervical cancer, WLHIV need to be regularly screened for cervical cancer (13).

Ethiopia adopted the WHO guideline and advised women to start cervical cancer screening at the age of 30–49 years at least one to three years intervals with the approach of seeing and treatment through visual inspection under acetic acid (12) as a screening strategy and cryo-therapy as a treatment method (14, 15). Only 0.6% of Ethiopian women aged 18 to 69 were checked for cervical cancer every three years, and evidence also showed that the incidence and prevalence of cervical cancer are increasing over time (16). In addition, many women in developing countries are diagnosed with cervical cancer at the late stages of the disease because of poor access to prevention options (17). This could be due to different factors including limited resources, there is a shortage of both opportunistic and organized population-based screening among HIV-positive women (18). Cervical cancer screening for WLHIV has just begun in a number of east Ethiopian hospitals. However data on the use of this service, particularly among HIV-positive eastern Ethiopian women, are lacking. Therefore, this study aimed to assess cervical cancer screening uptake and its associated factors among WLHIV in public hospitals in Harar, eastern Ethiopia.

## Methods and materials

### Study design and population

An institutional based cross-sectional study design was employed among WLHIV attending two public hospitals (Hiwot Fana Comprehensive Specialized University Hospital(HFCSUH) and Jugal Hospital), which provided antiretroviral therapy (3) and screening for cervical cancer in Harari Regional State Ethiopia. The study took place from March 20 to April 20, 2022.

### Sample size and sampling procedures

**Figure 1:**
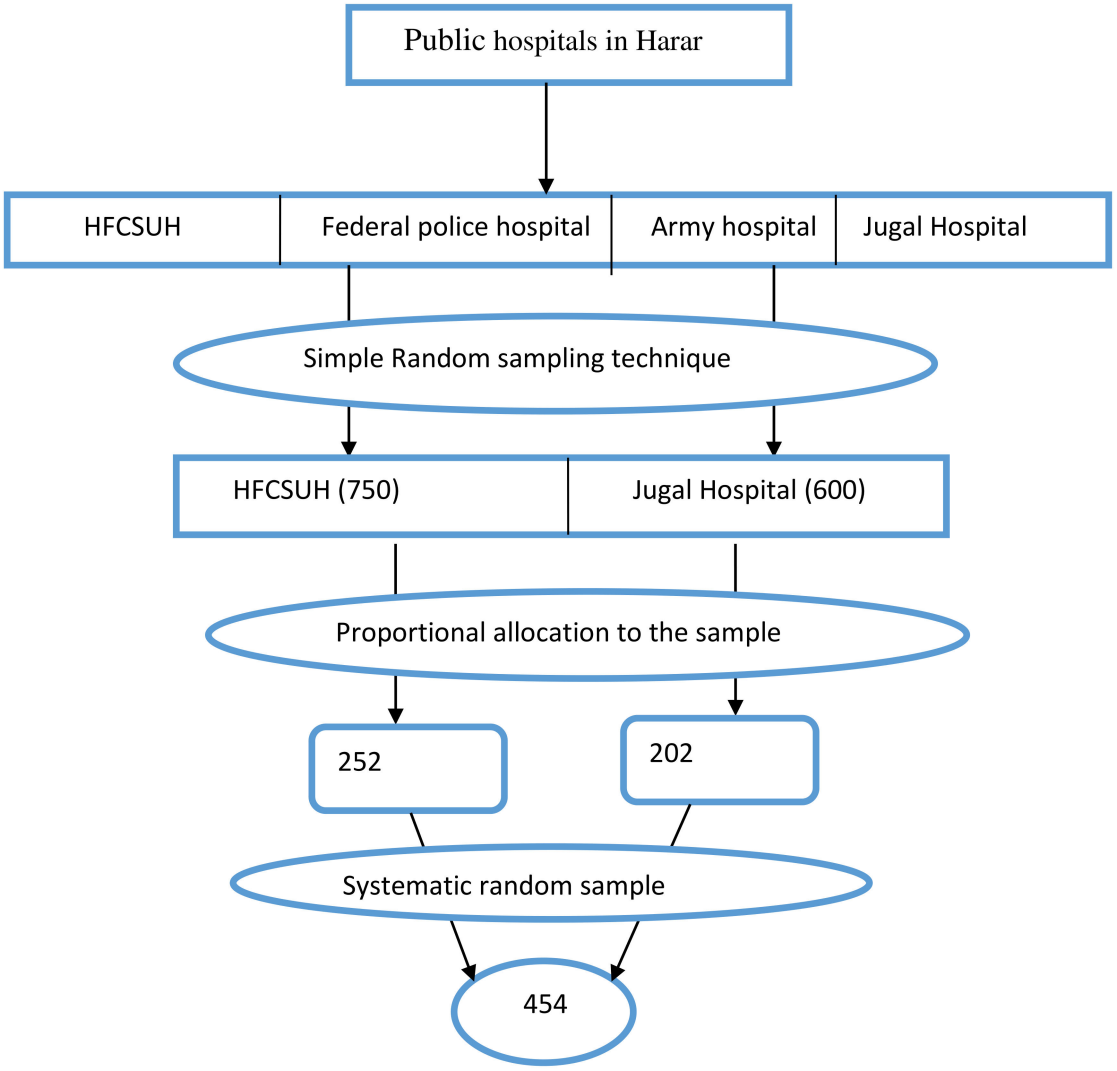
Schematic presentation of sampling procedure, Public Hospitals of Harar, Ethiopia.

The sample size was calculated using a single population proportion formula with the following assumptions: 95% confidence level, 4% margin of error, a prevalence of cervical cancer screening of 23.5% from previous study conducted in Northwest Ethiopia (19), and a 5% non-response rate. The final sample size was 454 participants. The sample was proportionally allocated between HFCUH and JH based on the ART client flow. Based on the inclusion criteria, study participants were selected using a systematic random sampling technique (**Figure 1**).

### Data collection methods and procedure

Data were collected using a structured interviewer -administered questionnaire adapted from the literatures (1, 10, 11, 20), which was written in English and translated into the local languages of Amharic, Oromo, and Harari, which were spoken by the majority of the study participants. A pre-test was performed before data collection. Four BSC nurses and two MSc nurses were involved in data collection and supervision after taking a two-day training. The principal investigator and supervisors collected the completed checklist and verified the completeness and consistency of the report. The study participants were approached by data collectors in the waiting area of the clinic as they waited to be attended by a healthcare provider. Information about the study was explained to the participants, including the procedures, potential risks, and benefits. After obtaining informed consent to participate in the study, the participants were interviewed using a questionnaire.

### Methods of data analysis

Responses to each questionnaire were coded for the simplicity of data entry. The coded data were then entered into Epi data 3.1 and exported to SPSS version 24 for data analysis. Data entry errors and missing values were checked and corrected before data analysis was conducted. In the first step, descriptive analyses such as percentages, frequency distribution, and measures of central tendency were computed to describe the study variables. A binary regression model was fitted to identify the associations between independent and outcome variables. Then, the assumptions of multicollinearity and outliers were checked, and a cutoff point of p<0.05 was significant for all the independent variables before fitting the model. All independent variables associated with the outcome variable (p-value < 0.25 and less in the bivariate analysis were entered into multivariable logistic regression models to identify independent predictors of cervical screening practice. Crude and adjusted odds ratios were computed with their corresponding 95% confidence intervals. Statistical significance was set at P < 0.05.

### Operational definitions

**Good knowledge;** Women who answered the knowledge questions and scored above the mean values were considered to have good knowledge.

**Uptake of cervical cancer screening**: WLHIV who were screened for premalignant cervical lesions at least once in their lifetime; otherwise, the women were considered as not uptake.

### Ethical consideration

Ethical clearance was obtained from the Haramaya University, College of Health and Medical Science, Institutional Health Research Ethics Review Committee (IHRERC) (Ref. No. IHRERC/039/2022). Official letters of cooperation were written to the HFCUH and JH to obtain their cooperation in facilitate this study. Information regarding the study was explained to the participants, including the procedures and benefits of the study. The respondents were informed of their right to refuse or decline participation in the study at any time, and their refusal to participate in the study did not affect them. Participants’ confidentiality was ensured by excluding the names and identifiers in the questionnaire. Informed voluntary, written, and signed consent was obtained from all respondents prior to the study.

## Results

### Socio-demographic characteristics of respondents

A total of 412 participants were enrolled in the study, and with response rate of 90.7%. The mean age of the respondents was 37.97 years (SD: 6.96) and 49.3% of them were between the ages between 30 and 39. Of the overall study participants, 46.8% were married, 53.9% had only primary education, and 82.3% were from urban areas (**Table 1**).

**Table 1:** Socio-demographic characteristics of WLHIV attending ART outpatients in public hospitals, Harar Eastern Ethiopia, 2022 (n=412).

Variable (n=412)	Category	Frequency	Percent
**Age in year**	20-29	40	9.7
	30-39	203	49.3
	40-49	142	34.5
	≥50	27	6.6
**Residence**	Urban	339	82.3
	Rural	73	17.7
**Religion**	Orthodox	219	53.2
	Muslim	128	31.1
	Protestant	65	15.8
**Occupation**	Housewife	114	27.7
	Self employed	101	24.5
	Employee in private organization	56	13.6
	government employee	82	19.9
	Daily laborer	55	13.3
**Monthly income (in birr($))**	≤500	35	8.5
	501-1500	87	21.1
	1501-2500	134	32.5
	≥2501	156	37.9
**Marital status**	Single	70	17.0
	Married	193	46.8
	Divorced	84	20.4
	Widowed	65	15.8
**Educational status**	No formal education	87	21.1
	Primary (1-8)	222	53.9
	Secondary (9-12)	88	21.4
	Tertiary education	15	3.6

### Cervical cancer screening up take

In this study, the overall uptake of cervical cancer screening was 57.5% (237/412) (95% CI: 52.5, 62.9). Of the respondents, 203 (49.3%) were aged 30-39 years, and only 63.5% of the 222 people with primary level education were screened for cervical cancer.

### Reason for not screening

**Figure 2:**
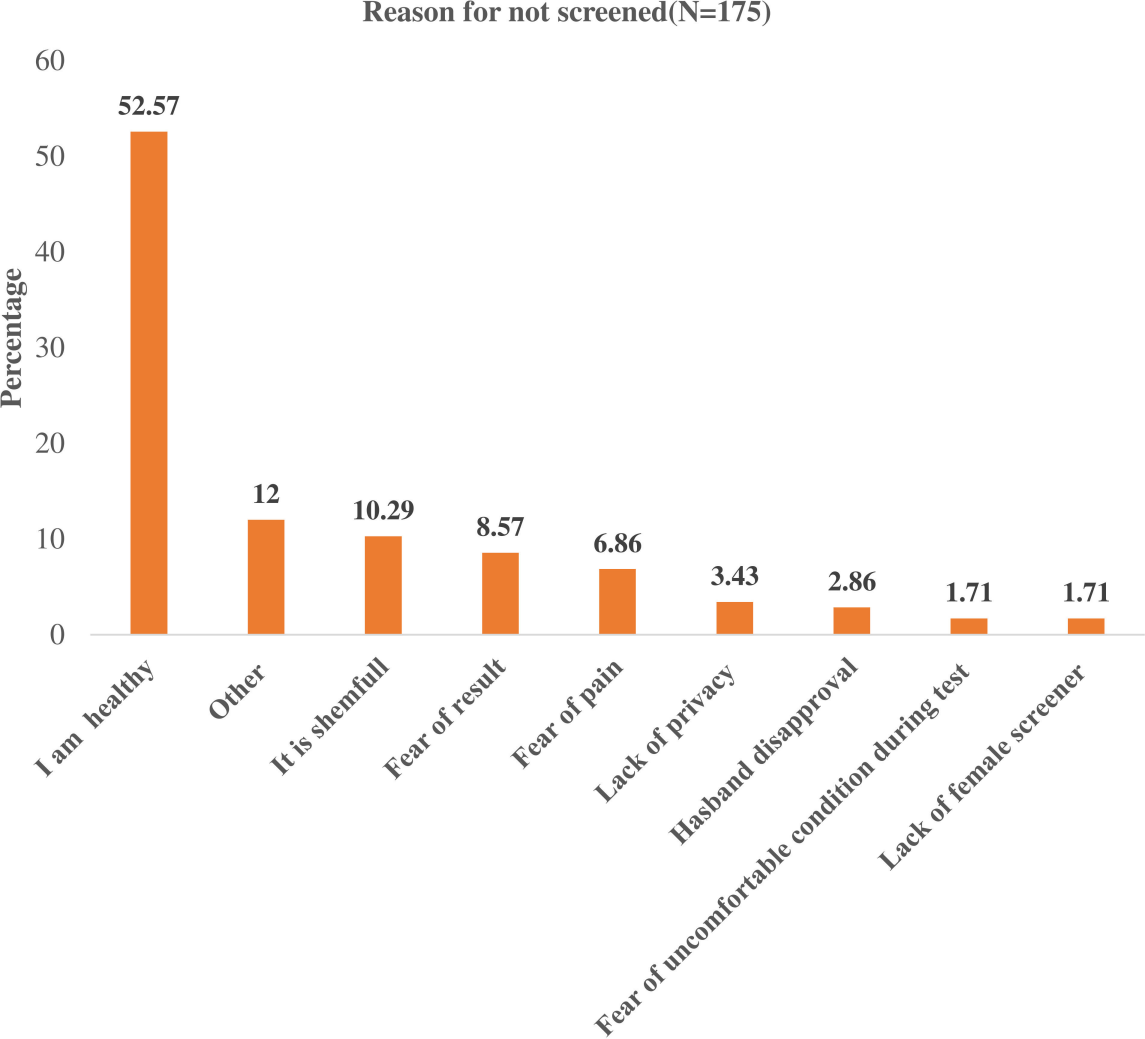
Reason for not screening of Cervical Cancer among Women Living with Human Immunodeficiency Virus in public hospitals, of Harar Eastern Ethiopia, 2022 (n=175).

Of the total number of respondents, 175 had never been screened for cervical cancer. The common reasons for not screening were feeling healthy (52.7%) followed by ashamed of screening (10.29%), and fear of the result (8.57%). Additional reasons for not screening were concerns about discomfort, loss of privacy, husband’s disapproval, and lack of time (**Figure 2**).

### Knowledge and awareness of the respondent on cervical cancer and screening

From a total of 412 respondents, about 370 (89.8%) had heard about cervical cancer screening, and 309 (75%) had good knowledge of the risk factors, signs and symptoms, and prevention. Among the respondents, 307 (74%) of them knew being HIV positive was risk for cervical cancer, the finding also revealed that 185 (44.9%) of the respondents knew the correct screening schedule for cervical cancer for HIV-positive women. The majority of respondents, 357 (86.7%) knew that cervical cancer was preventable. Of the respondents, 370 of them were volunteered to undergo for screening in the future and 57.5% of them had screened previously (**Table 2**).

**Table 2:** Respondents Knowledge and awareness on cervical cancer and screening of WLHIV in public hospitals, of Harar eastern Ethiopia, 2022.

Variable (n=412)	Category	Frequency	Percent
Have you ever heard about cervical cancer screening	No	42	10.2
	Yes	370	89.8
Have you ever been recommended by health worker	No	61	14.8
	Yes	351	85.2
Having multiple sexual partners is risk for cervical cancer	No	103	25.0
	Yes	309	75.0
Early age sexual intercourse is risk for cervical cancer	No	214	51.9
	Yes	198	48.1
Having an HPV infection is risk for cervical cancer	No	248	60.2
	Yes	164	39.8
Being HIV positive is risk for cervical cancer	No	105	25.5
	Yes	307	74.5
Vaginal discharge is sign of cervical cancer	No	186	45.1
	Yes	226	54.9
Vaginal bleeding is sign of cervical cancer	No	107	26.0
	Yes	305	74.0
Pain during coitus is sign of cervical cancer	No	214	51.9
	Yes	198	48.1
Cervical cancer is preventable	No	55	13.3
	Yes	357	86.7
Vaccination can prevent cervical cancer	No	322	78.2
	Yes	90	21.8
Screening prevents cervical cancer	No	138	33.5
	Yes	274	66.5
Cervical cancer is treatable	No	85	20.6
	Yes	327	79.4
Do you know where cervical cancer screening service is provided	No	48	11.7
	Yes	364	88.3
Have you ever done cervical cancer screening	No	175	42.5
	Yes	237	57.5
Do you know cervical cancer screening schedule	Every one year	185	44.9
	Every two year	61	14.8
	Don’t know	166	40.3
Would you undergo cervical cancer screening in the future	No	42	10.2
	Yes	370	89.8
Would you advise your relatives about cervical cancer screening	No	180	43.7
	Yes	232	56.3
Knowledge of the respondent	Poor	103	25.0
	Good	309	75.0

### Factors associated with cervical cancer screening

In the binary logistic regression, age, marital status, residence, education level, monthly income, and knowledge were significantly associated with cervical cancer screening uptake among adult WLHIV (p-value < 0.25) and were considered for multivariate analysis.

In the multivariable logistic regression, respondents age of–20-29 years and 40-49 years, tertiary level of education, monthly income, and knowledge were statistically significant at p-values less than or equal to 0.05.

Respondents aged between 20-29 years had 7.3 times the odds of being uptake cervical cancer screening compared with those aged > 50 years (AOR=7.33, 95% CI: 1.98, 27.1). And also, those age between 40-49 years had 4.37 times the odds of being uptake cervical cancer screening compared with those aged > 50 years (AOR=4.37, 95% CI: 1.48, 12.89). Those Participants who had tertiary level of education were 80.3% (AOR= 0.197 (95% CI: 0.041, 0.946)) less likely to undergo cervical cancer screening as compared to those who had no formal education. Respondents who had monthly income of 2501(45.52 $) and above Ethiopian birr were 61.1% less likely (AOR= 0.389 (95% CI: (0.158, 0.959)) to undergo cervical cancer screening as compared to those who had monthly income ≤500(9.10$) Ethiopian birrs. The odds of getting screened for cervical cancer among WLHIV having good knowledge were about 3.6 times (AOR= 3.591 (95% CI: 2.123, 6.073)) higher than those who had poor knowledge (**Table 3**).

**Table 3:** Bivariate and multivariable logistic regression analysis of factors associated with cervical cancer screening uptake among HIV-positive woman at public hospitals of Harar, eastern Ethiopia, 2022.

Variable	Category	Cervical cancer screening uptake		COR (95%CI)	AOR (95%CI)	P-value
		Yes	No			
**Age in year**	20-29	28 (70)	12 (30)	8.16 (2.6,25.3)	7.33 (1.9, 27.1)	0.003*
	30-39	113 (55.7)	90 (44.3)	4.39 (1.70-11)	2.78 (0.94,8.1)	0.062
	40-49	90 (63.3)	52 (36.6)	6.05 (2.3-15)	4.37 (1.48,12.89)	0.008*
	≥50	6 (22.2)	21 (77.8)	1	1	
**Residence**	Urban	201 (59.3)	138 (40.7)	1.497 (0.901,2.486)	1.080 (0.585,1.992)	0.806
	Rural	36 (49.3)	37 (50.7)	1		
**Marital status**	Single	50 (71.4)	20 (28.6)	1	1	
	Married	111 (57.5)	82 (42.5)	0.57 (0.300,0.979)	0.573 (0.298,1.100)	0.094
	Divorced	43 (51.2)	41 (48.8)	0.420 (0.214,0.822)	0.409 (0.192,0.872)	0.021
	Widowed	33 (50.8)	32 (49.2)	0.413 (0.203,0.840)	0.570 (0.240,1.353)	0.202
**Educational status**	No formal education	41 (47.1)	46 (52.9)	1	1	
	Primary	141 (63.5)	81 (36.5)	1.953 (1.182,3.226)	1.656 (0.901,3.046)	0.104
	Secondary	52 (59.1)	36 (40.9)	1.620 (0.891,2.947)	1.206 (0.555,2.620)	0.636
	Tertiary	3 (20)	12 (80)	0.280 (0.074,1.064)	0.197 (0.041,0.946)	0.042*
**Occupation**	Housewife	74 (64.9)	40 (35.1)	1	1	
	Self employed	51 (50.5)	50 (49.5)	0.551 (0.318,0.953)	0.707 (0.372,1.342)	0.289
	Employee in private organization	36 (64.2)	20 (35.7)	0.973 (0.499,1.898)	1.146 (0.525,2.503)	0.733
	Government employee	44 (53.7)	38 (46.3)	0.626 (0.350,1.118)	0.931 (0.428,2.023)	0.856
	Paid labor	31 (56.3)	24 (43.6)	0.698 (0.361,1.347)	0.996 (0.464,2.137)	0.992
	Other	1 (25)	3[1]	0.180 (0.181,1.789)	0.152 (0.013,1.745)	0.130
**Monthly income in birr**	≤500	23 (65.7)	12 (34.3)	1	1	
	501-1500	51 (58.6)	36 (41.3)	0.739 (0.326,1.674)	0.444 (0.174,1.129)	0.088
	1501-2500	83 (61.9)	51 (38.0)	0.849 (0.389,1.853)	0.445 (0.180,1.101)	0.080
	≥2501	80 (51.3)	76 (48.7)	0.549 (0.255,1.180)	0.389 (0.158,0.959)	0.040*
**Knowledge**	Good	201 (65.0)	108 (34.9)	3.463 (2.169,5.529)	3.591 (2.123,6.073)	0.000*
	Poor	36 (35.0)	67 (65.0)	1	1	

Where: 1=reference, * Significant at p-value of ≤0.05, AOR, Adjusted Odds Ratio.

## Discussion

In this study area about 57.5% (95% CI: 52.5, 62.9%) of WLHIV screened for cervical cancer, which implies a national target of addressing at least 80% of HIV-positive for cervical screening (21). Thus, cervical cancer screening services require strong collaboration between programmers and health care providers to accelerate service coverage among HIV-positive women. Age in years between 20-29, 40-49 years, tertiary level of education, knowledge, and monthly income were factors associated with cervical cancer screening uptake among HIV-positive women.

In this study, the uptake of cervical cancer screening uptake was 57.5%. This finding was comparable to that study conducted in Canada (58%) (22). But are higher than those reported in Hawasa Ethiopia (40.1%) (23), Northwest Ethiopia (23.5%) (19) Addis Ababa, Ethiopia (25.5%) (24), Nigeria (9%) (25), Morocco (9%) (26). However, this finding is lower than the study conducted in England (85.7%) (27) and Italy (91%) (12). The difference might be due to methodological variations, the sample collection period, differences in sociocultural, economic, health education, and awareness of health service utilization characteristics between respondents of the referenced areas and the study location during ART follow-up time, differences in access to reproductive health services, and community sensitization and awareness creation programs for cervical cancer and cervical cancer screening services. For instance, in Ethiopia, community mobilization and awareness creation through the expansion of urban and rural health extension programs have been implemented in recent years.

This research revealed that women’s knowledge of cervical cancer and screening is associated with screening uptake in this population. Women who were knowledgeable about cervical cancer and screening were approximately 3.6 times more likely to take up screening services than those who had poor knowledge. This result is supported by studies conducted in Tanzania (28), Botswana (29) and A systematic review in Ethiopia (30). This may be explained by the fact that the increase in women’s knowledge about cervical cancer and the benefits of screening directly lead them to utilize screening services.

In this study, when compared to individuals over the age of 50 years, those aged 20-29 had 7.3 times odds of cervical cancer screening. Furthermore, individuals aged 40-49 years had 4.37 times the odds of undergoing cervical cancer screening compared to those aged 50 years and older. This finding was supported by a study done in Southern Ethiopia (31, 32). A possible explanation for this might be that young age groups are productive and have a chance of getting more gynecological examinations, giving birth, and getting more health information about sexual and reproductive health, including cervical cancer, that promote screening services, so women of this age group might have better knowledge and intention to be screened.

The findings revealed that women’s educational level is associated with cervical cancer screening. In this study, women with tertiary-level education were less likely to undergo cervical cancer screening than those with no formal education. However, other studies conducted in India (33), Ghana (34) and Northwest Ethiopia (20) showed that as educational level increased, screening increased. A possible explanation could be that the number of participants in this level of education was low and the counselor had more influence on those with no formal education. Additionally, this study showed that among the respondents recommended by health care providers for screening, 66% of them were screened for cervical cancer, and this finding is comparable with the study conducted in Gurage zone, Southern Ethiopia (31). This could be expected even if the woman had no formal education counseling, and recommendations encouraged them to undergo screening.

In this study, women who had monthly income was significantly associated with cervical cancer screening. This finding shows that women’s monthly income 2501 and above Ethiopian birrwere 61% less likely to undergo cervical cancer screening as compared to women’s monthly incomes of 500 and above Ethiopian births. However, other studies have shown that women with low monthly income are less likely to engage in cervical cancer screening, and that lower economic status has a great impact on cervical cancer screening utilization (35). In fact, in Ethiopia, cervical cancer screening services are freely available; therefore, low monthly income women are more attracted to uptake the service, and having information about fee-free increases the uptake by low monthly-income women than others.

### Strengths and limitations of the study

This study was conducted on adult HIV-positive women, which may provide generalizable information to other parts of the community that use the services in other healthcare facilities. Furthermore, this study could be used as an input for future studies aimed at exploring the differences in cervical cancer screening uptake between HIV-positive and HIV-negative women. The possible limitations of this study include that Information that was self-reported by participants and the lack of a way to independently confirm the screening.

## Conclusion and recommendation

The rate of cervical cancer screening among WLHIV in this study was higher than that reported in a previous study conducted in Ethiopia. The respondents’ age, educational status, monthly income, and knowledge were significantly associated with cervical cancer screening utilization. To increase cervical cancer screening uptake, WLHIV should be counseled and promoted regarding the risk, prevention, and screening of cervical cancer, particularly those who attend ART follow-up. Other multidisciplinary techniques such as explaining the value of cervical cancer screening, education and capacity building, incorporating cervical cancer screening into community events, integrating cervical cancer screening into existing health services, create a dialogue on cervical cancer screening uptake among HIV positive women should be used to increase cervical cancer screening service utilization among women living with HIV.

## Data availability statement

The datasets presented in this study can be found in online repositories. The names of the repository/repositories and accession number(s) can be found in the article/supplementary material.

## Ethics statement

The studies involving humans were approved by Haramaya University, College of Health and Medical Science, Institutional Health Research Ethics Review Committee (IHRERC). The studies were conducted in accordance with the local legislation and institutional requirements. The participants provided their written informed consent to participate in this study.

## Author contributions

All authors made a significant contribution to the work reported, whether in the conception, study design, execution, acquisition of data, analysis, and interpretation, or in all these areas, took part in drafting, revising, or critically reviewing the article, gave final approval of the version to be published, agreed on the journal to which the article has been submitted, and agreed to be accountable for all aspects of the work.
